# Electrocardiographic Features of Left Ventricular Diastolic Dysfunction and Heart Failure With Preserved Ejection Fraction: A Systematic Review

**DOI:** 10.3389/fcvm.2021.772803

**Published:** 2021-12-17

**Authors:** Anne-Mar Van Ommen, Elise Laura Kessler, Gideon Valstar, N. Charlotte Onland-Moret, Maarten Jan Cramer, Frans Rutten, Ruben Coronel, Hester Den Ruijter

**Affiliations:** ^1^Laboratory of Experimental Cardiology, University Medical Center Utrecht, Utrecht, Netherlands; ^2^Julius Center for Health Sciences and Primary Care, University Medical Center Utrecht, Utrecht, Netherlands; ^3^Department of Cardiology, University Medical Center Utrecht, Utrecht, Netherlands; ^4^Department of Experimental Cardiology, Amsterdam University Medical Center, Amsterdam, Netherlands; ^5^Institut de rythmologie et modélisation cardiaque (IHU-Liryc), Pessac, France

**Keywords:** sex-differences, diagnosis, HFpEF-heart failure with preserved ejection fraction, LVDD-left ventricular diastolic dysfunction, primary care, electrocardiography (ECG)

## Abstract

**Background:** Electrocardiographic features are well-known for heart failure with reduced ejection fraction (HFrEF), but not for left ventricular diastolic dysfunction (LVDD) and heart failure with preserved ejection fraction (HFpEF). As ECG features could help to identify high-risk individuals in primary care, we systematically reviewed the literature for ECG features diagnosing women and men suspected of LVDD and HFpEF.

**Methods and Results:** Among the 7,127 records identified, only 10 studies reported diagnostic measures, of which 9 studied LVDD. For LVDD, the most promising features were T-end-P/(PQ^*^age), which is the electrocardiographic equivalent of the passive-to-active filling (AUC: 0.91–0.96), and repolarization times (QTc interval ≥ 350 ms, AUC: 0.85). For HFpEF, the Cornell product ≥ 1,800 mm^*^ms showed poor sensitivity of 40% (AUC: 0.62). No studies presented results stratified by sex.

**Conclusion:** Electrocardiographic features are not widely evaluated in diagnostic studies for LVDD and HFpEF. Only for LVDD, two ECG features related to the diastolic interval, and repolarization measures showed diagnostic potential. To improve diagnosis and care for women and men suspected of heart failure, reporting of sex-specific data on ECG features is encouraged.

## Introduction

The prevalence of heart failure with preserved ejection fraction is increasing relative to heart failure with reduced ejection fraction (HFrEF) (1), and affects women more than men in a 2:1 ratio (2). Left ventricular diastolic dysfunction (LVDD) is considered the pre-stage of heart failure with preserved ejection fraction (HFpEF). LVDD is marked by elevated filling pressures, abnormal relaxation, and decreased compliance of the left ventricle (LV), often accompanied by increased atrial volumes and left ventricular mass (3, 4). The lack of reliable diagnostic tools for the detection of HFpEF likely contributes to the underdiagnosis in primary care (5). Thus, direct referral for echocardiography follows when heart failure is suspected (6). Currently, echocardiography is not implemented in primary care, while ECG is. For HFrEF, certain ECG features are clearly linked, i.e., prolonged PR interval (7), low voltages (8), QRS prolongation (9), and QT prolongation, dispersion, and variability (10). Also, several ECG features were shown to be too help to identify HFrEF in primary care populations (11, 12). Similarly, ECG features could help in selecting patients needing echocardiography for HFpEF, but ECG features associated with HFpEF are less established. Recently, a meta-analysis reported a higher incidence of right bundle branch block (RBBB) or atrial fibrillation (AF) in HFpEF compared to HFrEF (13). This suggests that ECG changes associated with HFrEF cannot be directly extrapolated to HFpEF. However, in this meta-analysis, ECG features for LVDD were not studied and there was no comparison made with healthy individuals, or between women and men. Therefore, we performed a systematic review to identify ECG features in patients with LVDD or HFpEF. As the prevalence of HFpEF differs between men and women (2) and several ECG features are marked by sex-specific cut-offs (14), we also documented sex-specific reporting of diagnostic performance for LVDD and HFpEF.

## Methods

### Data Sources and Searches

We searched PubMed and EMBASE for articles on April 18, 2019 and updated our search up to October 26, 2021. Our search terms included electrocardiogram, diagnosis, heart failure, diastolic dysfunction, and variants of these terms and comprised only human studies. The full search string can be found in Supplementary Method I. After the removal of duplicates, all records were screened by title and abstract by two of three independent researchers (A.v.O., E.K., and G.V.). A further selection was made after reading full-texts and application of the in- and exclusion criteria. Disagreements were resolved by discussion. Among the studies retrieved for full-text assessment, reference lists were screened, and a citation search was performed for additional relevant studies by two researchers (A.v.O and E.K.).

### Study Selection

**Figure 1:**
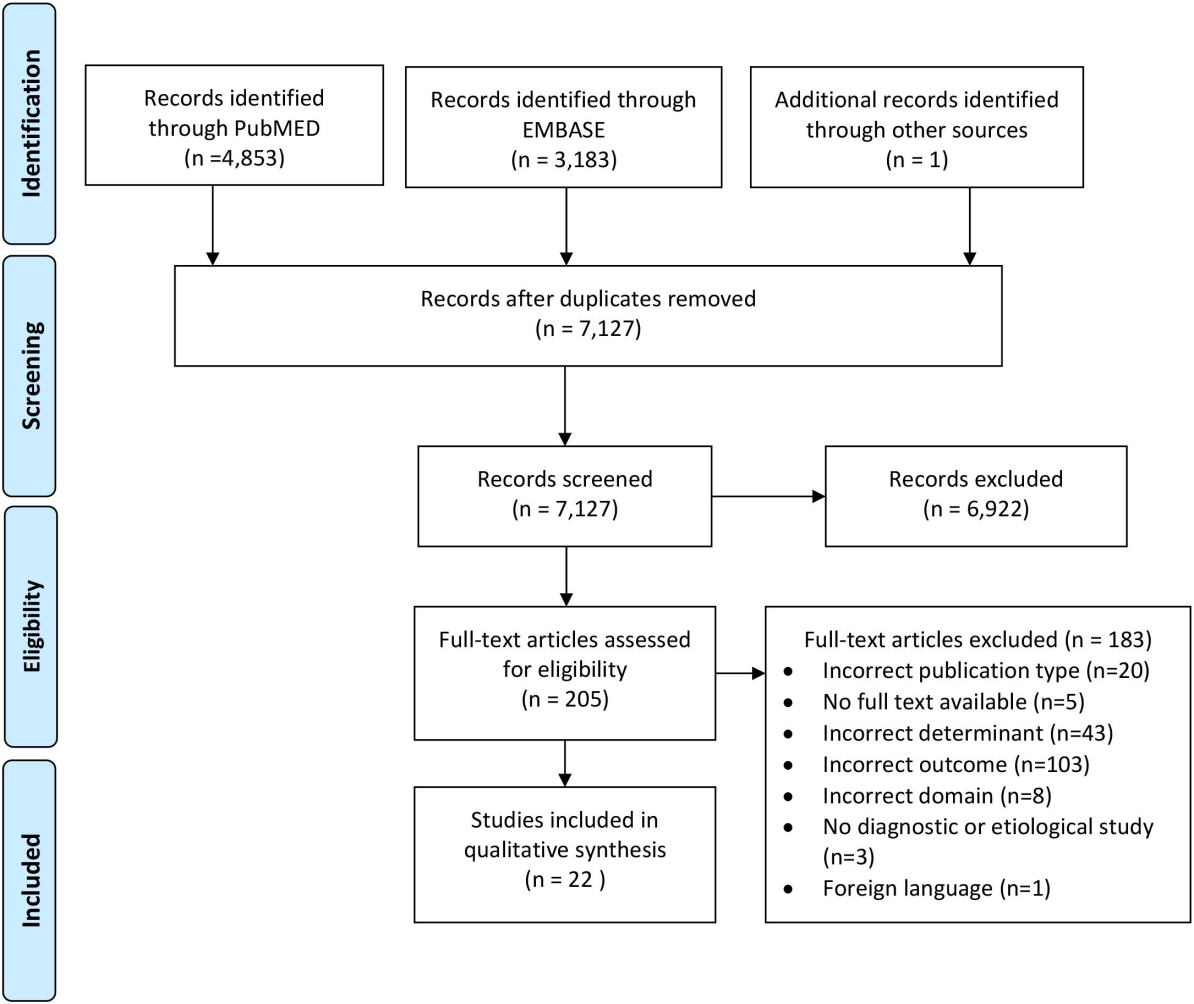
PRISMA flow diagram summarizing the search and selection process applying pre-defined in- and exclusion criteria.

Eligible studies were cross-sectional in patients suspected of LVDD or heart failure (domain), questioning whether ECG features (determinant) were diagnostic for LVDD or HFpEF (outcome). A 12-lead resting surface ECG should be part of the assessment. Participants should not have a history of the disease of interest, and the healthy controls were the non-diseased individuals as defined by the authors of the original articles. We excluded animal studies, *in vitro* studies, reviews, conference papers/abstracts, case studies, and editorials. For studies that were not full-text available, we contacted the corresponding author. If we did not receive a response, the study was excluded. Studies that were written in a language other than English, Dutch, or German were also excluded. Detailed information on well-defined ECG features had to be reported (e.g., exact values, cut-off values, or absence or presence of pre-defined criteria). Studies only reporting whether an ECG was normal or abnormal, without specifications, were not considered eligible. Diagnosis of LVDD or HFpEF had to be established according to existing guidelines (3, 4, 6, 15, 16). Studies on LVDD were only included if the diagnosis was based on multiple echocardiographic parameters to prevent misclassification (3, 16). The search and selection processes are visualized in the PRISMA flow diagram presented in Figure 1.

### Data Extraction

Study characteristics are reported in Supplementary Table I, including the name of the first author, year of publication, country, age and number of participants, percentage of women participating, study in- and exclusion criteria, mean left ventricular ejection fraction [LVEF (%)], ECG features studied, prevalence and definition of LVDD/HFpEF, and association measure between ECG feature and the diagnosis of LVDD or HFpEF. Additionally, we recorded if sex-stratified outcomes were given and whether sex was included in a multivariable model (if applicable). Data-extraction was performed by a single researcher (A.v.O.) and checked by another researcher (E.K.). We used the PRISMA reporting guidelines (17) and registered the protocol of this systematic review in PROSPERO (https://www.crd.york.ac.uk/prospero/) with the registration number: CRD42020212907.

### Critical Appraisal

For all studies selected, a critical appraisal was performed independently by two researchers (A.v.O, E.K.) in accordance with the QUADAS-2 criteria (18). Four domains i.e., patient selection, index test, reference test, and flow and timing were scored (Table 1). Additionally, the level of evidence in terms of the association measure provided for diagnosis of LVDD/HFpEF was rated. Studies presenting sensitivity/specificity/negative predictive value (NPV)/positive predictive value (PPV) and area under the curve (AUC) values were classified as the highest level of evidence. Odds ratio (OR), relative risk (RR), or correlation coefficient were classified as intermediate levels of evidence. Studies reporting numbers/percentages and between-group differences were judged as low level of evidence. As ECG parameters and association measures were highly heterogeneous, we only assessed publication bias when ≥5 studies reported the same ECG parameter and association measure. Based on the reported outcomes of the high level of evidence studies we judged ECG features as promising or not.

**Table 1:** Critical appraisal, evaluation of the level of evidence, and applicability for the selected studies in accordance with the QUADAS-2 criteria.

**Year of publication**	**1st author**	**Country/Population**	**Critical appraisal**				**Level of evidence**	**Applicability**		
			**Patient selection**	**Index test (ECG)**	**Reference test (Diagnosis)**	**Flow and timing**		**Domain**	**Determinant**	**Outcome**
2010	Boles	Ireland	Unclear	Low	Low	Low	Intermediate	No concerns	No concerns	No concerns
2003	Dogan	Turkey	Low	Low	Unclear	Low	Low	No concerns	No concerns	No concerns
2012	Eicher	France	Low	Unclear	Unclear	Low	Low	No concerns	No concerns	No concerns
2005	Gunduz	Turkey	High	Unclear	Unclear	High	Low	No concerns	No concerns	No concerns
2021	Hayiroglu	Turkey	Low	Unclear	Low	Low	High	No concerns	No concerns	No concerns
2012	Hsu	Taiwan	Low	Low	Low	Low	Intermediate	No concerns	No concerns	No concerns
2015	Kadi	Turkey	High	Low	Low	High	Intermediate	No concerns	No concerns	No concerns
2016	Khan	Pakistan	Unclear	Low	Unclear	Unclear	High	No concerns	No concerns	No concerns
2014	Krepp	USA	High	Low	Low	High	High	No concerns	No concerns	No concerns
2008	Miwa	Japan	High	Unclear	Unclear	Unclear	Low	No concerns	No concerns	No concerns
2013	Namdar	Switzerland	High	Low	Low	Unclear	High	No concerns	No concerns	No concerns
2018	Nikolaidou	UK	Low	Low	Low	Low	Low	No concerns	No concerns	No concerns
2012	Ofman	USA	High	Low	Unclear	High	Intermediate	No concerns	No concerns	No concerns
2016	Onoune	Japan	Unclear	Low	Low	Low	Intermediate	No concerns	No concerns	No concerns
2006	Palmieri	Europe/USA	Low	Low	Low	Low	Low	No concerns	No concerns	No concerns
2012	Sauer	USA	Low	Low	Low	Low	Intermediate	No concerns	No concerns	No concerns
2019	Sumita	Japan	Low	Unclear	Unclear	Low	High	No concerns	No concerns	No concerns
2014	Taha	Egypt	High	Low	Low	Low	High	No concerns	No concerns	No concerns
2019	Tan	Singapore	High	Unclear	Unclear	High	High	No concerns	No concerns	No concerns
2013	Tsai	Taiwan	Low	Low	Low	Low	High	No concerns	No concerns	No concerns
2011	Wilcox	USA	Low	Low	Low	Low	High	No concerns	No concerns	No concerns
2017	Yang	Australia	Low	Unclear	Unclear	Low	High	No concerns	No concerns	No concerns

*Green boxes represent either a low risk of bias, a high level of evidence, and no concerns with respect to applicability. Grey boxes represent an unclear risk of bias. Yellow boxes represent an intermediate level of evidence. Red boxes represent either a high risk of bias or a low level of evidence*.

## Results

**Figure 2:**
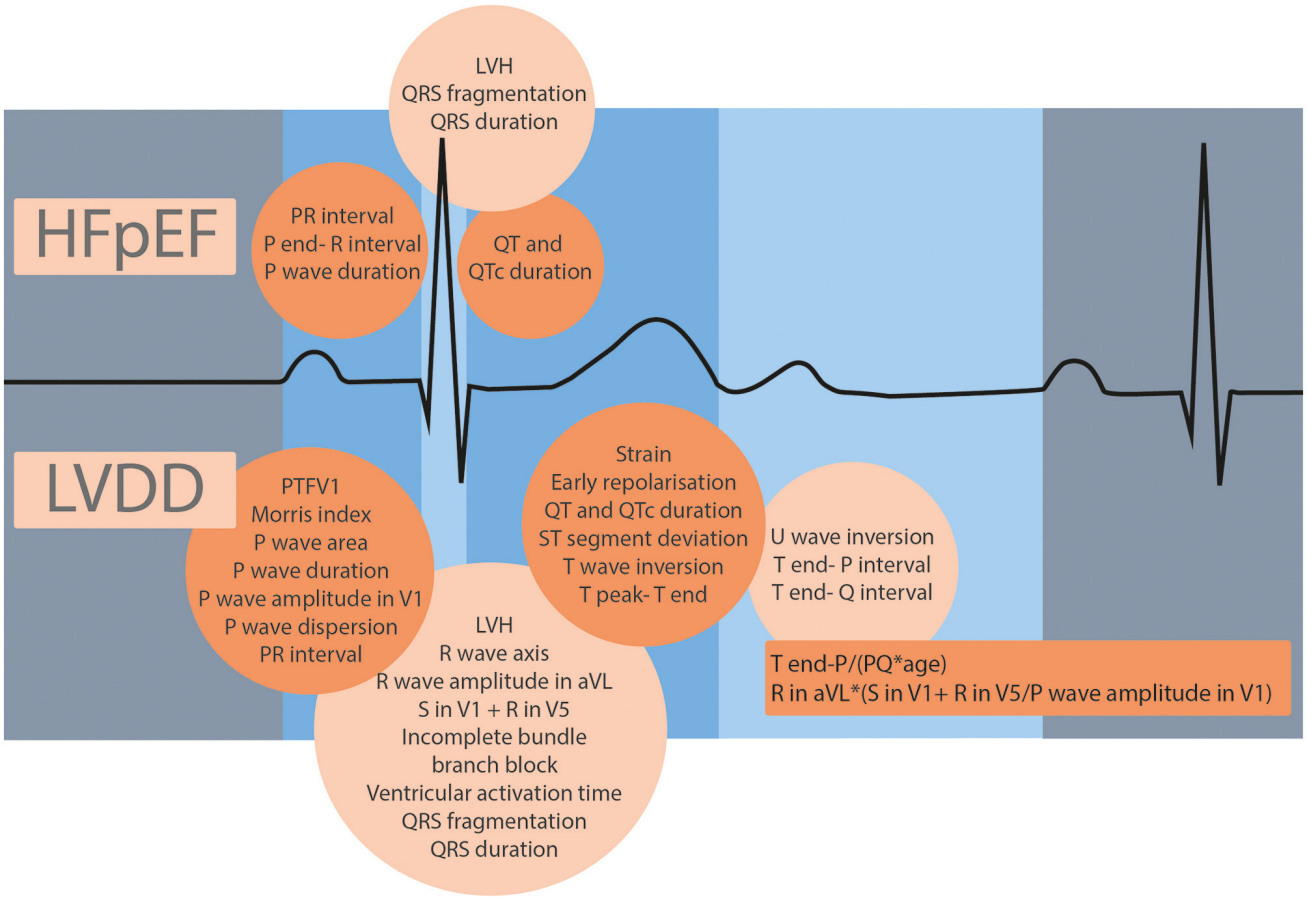
ECG features studied for HFpEF and LVDD, grouped by phase in the cardiac cycle.

In total, 7,127 articles were screened, and 22 met the predefined in- and exclusion criteria (Figure 1, Supplementary Table I). All 22 studies were published between 2003 and 2021. In total, 25 ECG parameters were investigated. Moreover, 16 parameters were studied only once. LVDD was the outcome in 18 studies and HFpEF in 4 studies. All 25 parameters were grouped by phase in the cardiac cycle: the atrial activation, ventricular depolarization, ventricular repolarization, and the full diastole (Figure 2, Supplementary Table II). All parameters from the 10 diagnostic studies are discussed in the text and summarized in Table 2.

**Table 2:** Summary of diagnostic association measures of ECG features for LVDD and HFpEF when compared to non-diseased individuals.

**LVDD/HFpEF**	**Phase**	**ECG feature**	**Definition**	**Study**	**Cut-off value**	**Findings**
	Atrial activation	P wave amplitude in V1	Peak of P wave to the iso-electric line of TP interval in lead V1	Hayiroglu et al. (19)	≥ 0.102 mV	AUC = 0.69, sensitivity = 67%, specificity = 60%
LVDD		PTFV1	P-wave terminal force in lead V1 is the multiplication of the amplitude by duration of the terminal part of the P-wave in lead V1.	Sumita et al. (20)	PTFV1 ≥0.04 mm*s	Sens = 27%, spec = 100%, PPV = 100%, NPV = 38%
				Yang et al. (21)	PTFV1 ≤ -4,000 μV*ms	Sens = 36%, PPV = 67%
		Morris Index	Present when P wave negative phase' width and amplitude are both > 1 mm.	Sumita et al. (20)		Sens = 13%, spec = 100%, PPV = 100%, NPV = 34%
		P wave area	P wave area is the multiplication of the P wave amplitude (mV) by 0.5 P wave duration (ms) in lead II.	Tsai et al. (22)	corrected P wave area > 60 ms*mV	AUC = 0.60, sens = 58%, spec = 56%
		P wave duration	Duration of P wave.	Tsai et al. (22)	corrected P wave duration > 85 ms	AUC = 0.62, sens = 65%, spec = 46%
				Sumita et al. (20)	P wave duration > 110 ms	Sens = 86%, spec = 86%
				Sumita et al. (20)	P wave duration > 120 ms	Sens = 34%, spec = 100%
		P wave dispersion	Difference between longest and shortest P wave recorded from multiple ECG leads.	Taha et al. (23)	P wave dispersion > 45 ms	Sens = 98%, spec = 64%
				Tsai et al. (22)	P wave dispersion > 65 ms	AUC = 0.62, sens = 62%, spec = 57%
		PQ- and PR interval	Beginning of P wave until onset of Q or R wave.	Namdar et al. (24)	PQ ≥ 150 ms	AUC = 0.65, sens = 78%, spec = 46%, PPV = 58%, NPV = 68%
	Ventricular depolarization	LVH	Most common criteria for left ventricular hypertrophy include: (1) Cornell voltage criteria: S in V3 + R in aVL > 28 mm (men), S in V3 + R in aVL > 20 mm (women). (2) Cornell product: (amplitude S in V3+R in aVL)*QRS duration. (3) Sokolow Lyon criteria: S wave in V1 and tallest R wave in V5 or V6 are ≥35 mm, or R wave in aVL ≥11 mm.	Krepp et al. (25)	Cornell product ≥ 1,595 mm*ms	Sens = 36%, spec = 90%, PPV = 83%, NPV = 52%
		Sum of S wave amplitude in V1 and R wave amplitude in V5		Hayiroglu et al. (19)	≥ 1.85 mV	AUC = 0.68, sensitivity and specificity = 65%
		R wave amplitude in aVL	R wave amplitude in aVL	Hayiroglu et al. (19)	≥0.517 mV	AUC = 0.68, sensitivity = 62%, specificity = 61%,
	Ventricular repolarization	QT interval	Interval between Q wave onset and end of T wave.	Taha et al. (23)	QT > 330 ms	Sens = 69%, spec = 64%
		QTc interval	As QT interval decreases when heart rate increases, QT interval is often corrected for heart rate (QTc) by Bazett's formula.	Taha et al. (23)	QTc ≥ 395 ms	Sens = 81%, spec = 79%
				Khan et al. (26)	QTc ≥ 435 ms	AUC = 0.82, sens = 71%, spec = 81%, PPV = 65%, NPV = 85%
				Wilcox et al. (27)	QTc ≥ 435 ms	Sens = 73%, spec = 74%
		ST segment deviation	ST segment deviation from J point of at least 20 mV.	Yang et al. (21)		Sens = 28%, PPV = 67%
		T peak—T end	Interval between peak and end of T wave.	Taha et al. (23)	T peak—T end > 95 ms	Sens = 76%, spec = 29%
	Full diastolic period	T end—P interval	End of T wave to P wave onset.	Namdar et al. (24)	T end—P ≤ 311 ms	AUC = 0.82, sens = 79%, spec = 72%, PPV = 74%, NPV = 78%
		T end—Q interval	End of T wave to Q wave onset.	Namdar et al. (24)	T end—Q ≤ 455 ms	AUC = 0.77, sens = 73%, spec = 73%, PPV = 73%, NPV = 73%
	Indexes	T end-P/(PQ*age)		Namdar et al. (24)	(T end-P/(PQ*age) ≥ 0.0333	AUC = 0.96, sens = 90%, spec = 92%, PPV = 91%, NPV = 90%
		T end-Q/(PQ*age)		Namdar et al. (24)	(T end-Q/(PQ*age) ≥ 0.0489	AUC = 0.95, sens = 89%, spec = 94%, PPV = 94%, NPV = 90%
		R in aVL * (S in V1 + R in V5)/P wave amplitude in V1)		Hayiroglu et al. (19)	≥ 8.53 mV	AUC = 0.78, sensitivity and specificity = 70%
HFpEF	Ventricular depolarization	LVH	See LVDD	Tan et al. (28)	Cornell product ≥ 1,800 mm*ms	AUC = 0.62, sens = 40%, spec = 80%

*AUC, area under the receiver operating characteristics curve; BBB, bundle branch block; HFpEF, heart failure with preserved ejection fraction; LVDD, left ventricular diastolic dysfunction; NPV, negative predictive value; PPV, positive predictive value; PTFV1, P-wave terminal force in lead V1; LVH, left ventricular hypertrophy; sens, sensitivity; spec, specificity*.

### Critical Appraisal

The overall quality of the studies was acceptable, all studies met the applicability criteria, and six studies had an overall low risk of bias on all domains (Table 1). We did not exclude studies because of a high risk of bias. The major reason for the high risk of bias in the study selection domain was a case-control design. Secondly, many studies applied extensive exclusion criteria that led to the exclusion of difficult to diagnose patients affecting the diagnostic accuracy of ECG features and reducing the generalizability of the findings. Information on blinded interpretation of the index test and reference was often lacking resulting in an unclear risk of bias in these domains. The interval between performing the ECG and the echocardiogram (assessed in the flow and timing domain) was often not reported, but no stringent concerns were raised in this period was longer than 6 weeks. The majority of studies had a low or intermediate level of evidence. A total of nine studies reported appropriate association measures for the diagnosis of LVDD or HFpEF and were thus classified as a high level of evidence.

### Atrial Contraction Related Features

Electrocardiographic (ECG) features derived from atrial contraction up to the ventricular depolarization were described in 11 articles (20–25, 29–33).

#### PTFV1 and Morris Index

In 417 individuals considered at risk for heart failure (e.g., history of hypertension, diabetes, obesity, or having received potential cardiotoxic chemotherapy) enrolled through local media advertising, the P-wave terminal force in lead V1 (PTFV1) ≤ −4,000 μV^*^ms showed a PPV of 67% and a sensitivity of 36% for LVDD (prevalence LVDD = 65%) (21). In another study with individuals undergoing echocardiography as part of routine cardiac care (20), the sensitivity, specificity, PPV, and NPV of a PTFV1 ≥0.04 mm^*^s were 27, 100, 100, and 38%, respectively, for a diagnosis of LVDD [present in 62 of 117 participants (53%)]. In 8 among the 117 participants (6.8%), the Morris index was present resulting in a sensitivity, specificity, and PPV and NPV for LVDD of 13, 100, 100, and 34%, respectively (20).

#### *P*-Wave Area, Dispersion, and Duration

In 140 individuals in whom coronary artery disease (CAD) was ruled out with a negative exercise test or coronary angiography (CAG), *P*-wave dispersion (>0.045 s) showed a sensitivity and specificity of 98 and 64% for LVDD (prevalence LVDD = 60%) (23). In another study in 270 patients undergoing echocardiography for clinical indications (e.g., abnormal physical examination, hypertension, or suspicion of CAD or heart failure), *P*-wave duration, *P*-wave area, and dispersion were measured (22). Measurements were corrected for heart rate using Bazett's formula, and for all features, significantly higher values were found in individuals with LVDD compared to those without LVDD (prevalence LVDD = 33%). For the corrected *P*-wave area, the AUC for diagnosing LVDD was 0.6 (22). The AUC for both corrected *P*-wave duration, and *P*-wave dispersion was 0.62. In a similar population (prevalence LVDD = 53%), *P*-wave duration > 110 ms was more sensitive for LVDD (sensitivity 86%, specificity 86%), and a *P*-wave duration > 120 ms was more specific for LVDD (sensitivity 34% and specificity 100%) (20).

#### *P*-Wave Amplitude

The amplitude of *P*-wave was measured in one study with LVDD as an outcome in 204 individuals without CAD or other major cardiac pathologies visiting the outpatient cardiology clinic (19). At a cut-off value ≥ 0.102 mV, this parameter showed a sensitivity of 67% and specificity of 60% with an AUC of 0.69 in this population with a prevalence of LVDD of 42%.

#### PQ Interval

One study reported the diagnostic performance of a PQ interval of ≥ 150 ms for LVDD, in individuals with diastolic function classification based on echocardiography (24). AUC, sensitivity, specificity, PPV and NPV were 0.65, 78, 46, 58, and 68%. In this study, LVDD was present in 81 of the 164 participants (prevalence = 49%).

### Ventricular Depolarization

In total, 9 studies reported ECG parameters representing ventricular depolarization and their relationship to LVDD (21, 24, 25, 28, 29, 33–36). Of note, many studies (19, 20, 23, 25, 28, 35, 37) used a QRS duration of above 120 or 130 ms, or the presence of complete bundle branch block (BBB), as exclusion criteria.

#### Left Ventricular Hypertrophy

The Cornell product with a cut-off value ≥ 1,595 mm^*^ms based on the 3rd quartile Cornell product was used to determine LVDD (prevalence = 57%) in a group of 185 individuals, undergoing both echocardiography and coronary computed tomography angiography (CCTA) for clinical indications (25). For the detection of LVDD, the sensitivity and specificity were 36 and 90% and PPV and NPV were 83 and 52%, respectively. Another study used 3rd quartile sex-specific cut-off values of the Cornell product (1,442 mm^*^ms for men and 1,515 mm^*^ms for women) and found a PPV and sensitivity of 77 and 29% for LVDD (prevalence LVDD = 65%) (21).

In the only study reporting diagnostic association measures for HFpEF, a Cornell product ≥ 1,800 mm^*^ms showed a sensitivity, specificity, and AUC of 40, 80, and 0.62 for the detection of HFpEF (prevalence HFPEF = 52%) when compared to controls with hypertension (28).

Another group used the sum of the amplitude in S wave in V1 and R wave in V5 (derived from the Sokolow-Lyon criteria) as a diagnostic measure for LVDD in individuals without CAD or other major cardiac pathologies (19). This ECG feature showed a sensitivity of 62%, specificity of 61%, and AUC of 0.68 at a cut-off value of ≥ 1.85 mV. The same authors also studied R wave amplitude in lead aVL. For this feature, lower sensitivity and specificity of 60%, and AUC of 0.65 were found at a cut-off of ≥0.517 mV.

### Ventricular Repolarization

Features of ventricular repolarization, defined as the period between the end of the QRS complex and the end of the *T*-wave, were reported by 12 studies (21, 23–27, 33, 37–39).

#### QTc and QT Interval

In 140 individuals without signs of CAD (based on stress ECG or CAG), QT and QTc intervals were significantly longer in individuals with LVDD compared to individuals without LVDD (prevalence LVDD = 60%) (23). A QTc interval ≥ 395 ms could diagnose LVDD with a sensitivity and specificity of 81 and 79%, whereas a QT interval > 330 ms showed lower sensitivity and specificity of 69 and 64%, respectively. Wilcox et al. measured QTc interval, QT interval, and J point- T interval corrected for heart rate (JTc) is firstly a derivation group referred for the suspicion of heart failure, and secondly, a validation group referred for stress echocardiography (prevalence LVDD = 64% in the derivation group) (27). For the detection of grade II or higher LVDD in the derivation group, a QTc interval ≥ 435 ms had a sensitivity and specificity of 73 and 74%. A QTc interval ≥435 ms in the validation cohort was associated with lower e' velocities, but diagnostic association measures for LVDD categories were not reported. For both the derivation and validation groups QT intervals were higher in individuals with LVDD, but diagnostic association measures were not reported. A significant interaction between JTc interval and QRS duration was observed, however, there was no significant association between JTc and a reduced septal e' velocity in individuals with prolonged QRS duration. One other study, with LVDD as the outcome (prevalence LVDD = 60%), used the same cut-off value for QTc duration and found sensitivity, specificity, NPV, PPV, and AUC value of 71, 81, 85, 65%, and 0.82, respectively, in 300 individuals with the suspicion of heart failure (26).

#### ST-Segment Deviation

In a group of patients at risk for heart failure, ST-segment deviation in lead V5 and V6 was present in 29% compared to 25% of the participants with and without LVDD (prevalence LVDD = 65%). PPV and sensitivity for LVDD were 67 and 28%, respectively (21). Individuals with known CAD were excluded in this study, but the presence of CAD in the study population was not stated.

#### T-Peak-T-End Interval

In 140 individuals where CAD was ruled out, there was no significant difference for T-peak-T-end interval comparing individuals with and without LVDD. Sensitivity and specificity were 76% and 29%, respectively (23).

### Diastolic Period and Indexes

The diastolic period, defined as the end of the T-wave until the onset of the QRS complex, comprised two studies (24, 40).

#### Indexes Related to Diastolic Period: T-End-P/(PQ^*^age) and T-End-Q/(PQ^*^age)

A study in 164 individuals with echocardiography data available on LVDD classification (24) found that T-end-P-interval and T-end-Q-interval were significantly shorter in individuals with LVDD compared to without LVDD. Two diagnostic indexes consisting of several ECG features and age were tested in the derivation group of this study, the first index being T-end-P/(PQ^*^age), the second being T-end-Q/(PQ^*^age). The first index showed an AUC value of 0.96 and sensitivity, specificity, PPV, NPV, and accuracy of above 0.9 for LVDD at a cut-off value of 0.0333. As a reference, the value of this index was 0.06 ± 0.026 for individuals ≤ 60 years without LVDD, compared to 0.0269 ± 0.005 for individuals in this age category with grade II LVDD (*p* < 0.005). For individuals, > 60 years old without LVDD a value of 0.042 ± 0.011 was found, compared to 0.021 ± 0.01 in grade II LVDD. Similarly, the AUC for the second index was high at 0.95 with high sensitivity, specificity, PPV, NPV, and accuracy for LVDD at a cut-off value of 0.0489. The index T-end-P/(PQ^*^age) was also validated reporting an AUC value of 0.91 and high values for sensitivity, specificity, PPV, NPV, and accuracy (82, 93, 93, 82, and 88%, respectively).

#### Electrocardiographic Diastolic Index (EDI)

In a study of 204 patients without CAD, or other major cardiac pathologies the validity of an ECG index involving *P*-wave amplitude in lead V1, components of the Sokolow-Lyon criteria, and Cornell product was tested. The index being aVL R wave amplitude ^*^ (V1 S amplitude + V5 R amplitude)/P wave amplitude in V1) showed the highest diagnostic value for LVDD when the index was ≥ 8.53 mV with an AUC of 0.78, the sensitivity of 70%, and specificity of 70%.

### ECG Cut-Off Values and Outcomes in Women and Men

None of the studies reported diagnostic properties of ECG features separately for women or men. However, Yang et al. used sex-specific cut-off values for the Cornell product (21). Although sex-specific outcomes were not reported, many intermediate levels evidence studies performing multivariate regression analysis used biological sex as a covariate (21, 22, 27, 28, 37, 38).

## Discussion

Electrocardiographic (ECG) features of LVDD and HFpEF were not frequently studied, and we identified 8 studies that showed diagnostic performance of ECG features in LVDD. Only one study reported the diagnostic value of ECG features in HFpEF. No studies reported data for women and men separately despite known differences between men and women in prevalence of HFpEF, and in normal electrocardiographic times.

### Discussion of the Different Identified Features

The index [T-end-P/(PQ^*^age)], which electrocardiographically reflects the ratio of the early filling phase to the atrial contraction phase of the diastole, showed a reduced ratio with worsening diastolic function. This index, described by Namdar et al. (24) showed the best diagnostic properties (AUC:0.96 and 0.91 in the derivation and validation group) of all ECG features studied. It showed that it was able to identify LVDD in situations, where echocardiography is not directly available. This index has not yet been validated further.

As the early filling phase (T-end-P) shortens when QT and PQ intervals are prolonged and heart rate increases, it is not surprising that many studies reported the association of higher PQ and QTc intervals with LVDD (13, 20, 22–27, 30, 32). PQ time, as well as *P*-wave dispersion and duration, have been established as markers of cardiac degeneration and as risk factors for atrial fibrillation and all-cause mortality (41). Biphasic *P*-waves are typically associated with dilated atria in heart failure and a negative force in lead V1 is mandatory for abnormal PTFV1 and the Morris index. The association of increased atrial conduction times with LVDD and HFpEF underlines the idea that LVDD and HFpEF are outcomes of accelerated cardiac aging (42).

The QTc interval is longer in women compared to men (14, 43), and therefore has sex-specific cut-off values (44). The QTc interval can be influenced by many factors, e.g., genetic disorders, medication usage, electrolyte disorders, obesity, diabetes, and a prolonged QRS duration (44). Although QTc prolongation observed in LVDD is not explained by prolonged QRS duration as shown by Wilcox et al. (27), left ventricular myocardial systolic and diastolic dyssynchrony has been observed in HFpEF patients with narrow QRS complexes when compared to healthy controls (45). Hypothetically, this dyssynchrony could be driven by altered intracellular calcium handling in cardiomyocytes, a condition that also can result in QTc prolongation (46). Alternative explanations for QTc prolongation in LVDD could be an autonomic imbalance (42, 47), or influences of comorbidities and medication usage, although some of the studies in this review excluded individuals using QTc prolongation medication (23, 30).

Although an increased left ventricular mass index is part of the structural domain within the HFA-PEFF algorithm (4) for HFpEF diagnosis, the poor diagnostic performance of electrocardiographic signs of LVH was described, for both LVDD and HFpEF. Hayiroglu et al. (19) tested an index predominantly involving amplitude signals for LVH, and P wave amplitude, as a measure for LVDD based on the hypothesis that these signals are predictive for LVDD given the high prevalence of LVH and AF in this population. Criteria related to slower ventricular conduction were deliberately left out of the equation because the authors reasoned these are predictive of CAD and HFrEF. However, this index had poorer diagnostic performance compared to the [T-end-P/(PQ^*^age)] index.

### Heterogeneity in Determinants and Association Measures

There is large heterogeneity in the (cut-offs of) ECG features that were reported in the different studies, which resulted in a small number of studies that investigated the same ECG feature. Also, some studies corrected ECG features for heart rate, while others did not. As deconditioning and autonomic imbalance in heart failure generally leads to higher resting heart rates (48), the usefulness of heart rate correction in HFpEF diagnosis is controversial and worth investigating.

We only selected studies that diagnosed LVDD or HFpEF in line with current or prior guidelines, but as the diagnostic criteria considered the gold standard changed frequently over the years, this resulted in the heterogeneity of assessment of LVDD and HFpEF (3, 4, 6, 15, 16).

Many studies did not report the diagnostic properties of the parameters studied, leading to a low level of evidence. However, when diagnostic properties were provided, there was also heterogeneity in the diagnostic properties described. For example, only reporting PPV and sensitivity (21), leaves question marks about the discriminative value of the ECG features studied. Altogether, this resulted in limited comparability of the included studies. Thus, it was not possible to pool studies in a meta-analysis, nor to assess publication bias. Nevertheless, some of the low levels of evidence studies showed neutral results comparing individuals with LVDD and HFpEF to controls (Supplementary Table I).

### Strengths and Limitations

Strengths: We addressed the value of ECG features in diagnosing LVDD and HFpEF in a systematic manner. In addition, we reported if and how sex is accounted for in the analyses, which is important to identify knowledge gaps that currently still exist in the field of cardiology.

Limitations: We included only studies with a 12-lead resting surface ECG. Hence, we excluded studies that took features from exercise ECGs such as heart rate variability and ST-segment hump sign (23, 47, 49, 50). We recognize that those may be relevant for the diagnosis of LVDD and HFpEF, but interpretation and implementation in primary care would be a limitation.

### Recommendations and Directions for Future Research

Both features that showed high diagnostic performance for LVDD, the index reflecting the ratio of passive and active filling and ventricular repolarization times, were not studied in HFpEF. We recommend validation of these features for HFpEF in individuals suspected of heart failure, taking into account specific conditions such as premature ventricular beats or drug regiments. In addition, we recommend that future studies based on implementation reports on the inter-observer performance of ECG features be studied and assess whether measuring ECG features needs training. ECG features for LVDD and HFpEF diagnosis could be very useful in primary care, but the interpretation by healthcare workers with limited experience in reading ECGs could decrease applicability. Although more complex, many efforts are undertaken to produce reliable (screening) methods using deep learning algorithms for LVDD and HFpEF diagnosis (51–54). The largest potential of these models is adding features distilled from raw ECG data that would otherwise not be accessible, thus providing new information. Finally, we recommend disclosing how ECG features for LVDD and HFpEF perform in men and women separately to increase application in clinical practice.

## Conclusion

Electrocardiographic (ECG) features are not widely evaluated in diagnostic studies for LVDD and HFpEF. Only for LVDD, two ECG features related to the diastolic interval, and repolarization measures showed diagnostic potential. To improve diagnosis and care for women and men suspected of heart failure, reporting of sex-specific data on ECG features is encouraged.

## Data Availability Statement

The original contributions presented in the study are included in the article/Supplementary Material, further inquiries can be directed to the corresponding author/s.

## Author Contributions

The literature search, data collection, and analysis were performed by GV, EK, and A-MV. The first draft of the manuscript was written by A-MV and EK. All authors commented on previous versions of the manuscript, contributed to the study conception and design, and read and approved the submitted version.

## Funding

This research was funded by European Research Council consolidator grant 866478 (UCARE), Dutch Cardiovascular Alliance grant 2020B004 (IMPRESS), Leducq Network of Excellence 16CVD02 (RHYTHM), and Dutch Cardiovascular Alliance grant 2020B008 (RECONNEXT).

## Conflict of Interest

The authors declare that the research was conducted in the absence of any commercial or financial relationships that could be construed as a potential conflict of interest.

## Publisher's Note

All claims expressed in this article are solely those of the authors and do not necessarily represent those of their affiliated organizations, or those of the publisher, the editors and the reviewers. Any product that may be evaluated in this article, or claim that may be made by its manufacturer, is not guaranteed or endorsed by the publisher.
