# Supplementary material for: Electrocardiographic Features of Left Ventricular Diastolic Dysfunction and Heart Failure With Preserved Ejection Fraction: A Systematic Review
Source: Front Cardiovasc Med. 2021 Dec 17;8:772803. doi: 10.3389/fcvm.2021.772803 (PMC8719440; doi:10.3389/fcvm.2021.772803)
Supplement: Supplementary file 1 [file Data_Sheet_1.pdf]

Supplemental Materials

Supplemental Method I

Supplemental Table I

Supplemental Table II

Supplemental References

Supplemental Method I:

Search string PubMED (4564 records), language filters: German; English; Dutch

((((((((((Electro cardiogr*[Title/Abstract]) OR Elektro cardiogr*[Title/Abstract]))) OR ((Electrocardiogr*[Title/Abstract]) OR Elektrocardiogr*[Title/Abstract])) OR "Electrocardiography"[Mesh])) OR (ecg[Title/Abstract] OR ekg[Title/Abstract]))) AND ((("Heart Failure"[Mesh:noexp]) OR ((heart failure[Title/Abstract]) AND ((diastolic[Title/Abstract]) OR (preserved ejection fraction[Title/Abstract]) OR (pef[Title/Abstract])))) OR (("Ventricular Dysfunction"[Mesh]) AND (diastolic[Title/Abstract])) OR ((diastolic dysfunction[Title/Abstract]) OR (lvdd[Title/Abstract])) OR ((failure[Title/Abstract] OR decompensation[Title/Abstract] OR insufficiency[Title/Abstract] OR dysfunction[Title/Abstract] OR disfunction[Title/Abstract]) AND (ventricular[Title/Abstract] OR cardiac[Title/Abstract] OR heart[Title/Abstract] OR myocardial[Title/Abstract]) AND (diastolic[Title/Abstract]))))) NOT ((animals[mesh] NOT humans[mesh])) NOT (cardiomyopathies [mesh] OR cardiomyopath* [Title/Abstract]OR takotsubo cardiomyopathy[mesh] OR takotsubo [Title/Abstract]) NOT (“cardiac pacing, artificial” [mesh]) NOT (“pacemaker, artificial” [mesh]) NOT (“defibrillators, implantable” [mesh]) NOT (“Clinical Protocols” [mesh]) NOT (“research design” [mesh]) NOT (“Drugs, Investigational” [mesh]) NOT (“Cardiovascular Surgical Procedures” [mesh] OR “Minimally Invasive Surgical Procedures” [mesh]) NOT (“drug therapy” [mesh]) NOT (review [Title/Abstract] OR meta-analysis [Title/Abstract])

Search string EMBASE (2585 records)

('electro cardiogr*':ab,ti,kw OR 'elektro cardiogr*':ab,ti,kw OR electrocardiogr*:ab,ti,kw OR elektrocardiogr*:ab,ti,kw OR 'electrocardiography'/exp OR ecg:ab,ti,kw OR ekg:ab,ti,kw) AND ('heart failure'/de OR ('heart failure':ab,ti,kw AND (diastolic:ab,ti,kw OR 'preserved ejection fraction':ab,ti,kw OR pef:ab,ti,kw)) OR ('heart ventricle function'/exp AND diastolic:ab,ti,kw) OR 'diastolic dysfunction':ab,ti,kw OR lvdd:ab,ti,kw OR ((failure:ab,ti,kw OR decompensation:ab,ti,kw OR insufficiency:ab,ti,kw OR dysfunction:ab,ti,kw OR disfunction:ab,ti,kw) AND (ventricular:ab,ti,kw OR cardiac:ab,ti,kw OR heart:ab,ti,kw OR myocardial:ab,ti,kw) AND diastolic:ab,ti,kw)) NOT ([animals]/lim NOT [humans]/lim) NOT (cardiomyopath*:ab,ti,kw OR cardiomyopath*/exp OR takotsubo:ab,ti,kw OR 'takotsubo cardiomyopathy'/exp) NOT (pacing:ab,ti,kw OR 'heart pacing'/exp) NOT (pacemaker:ab,ti,kw OR 'cardiac rhythm management device'/exp) NOT (defibrillator*:ab,ti,kw OR 'defibrillator'/exp) NOT ('device safety'/exp) NOT (‘Clinical Protocol*’:ab,ti,kw OR 'clinical protocol'/exp) NOT (‘research design’:ab,ti,kw) NOT ('drug therapy':ab,ti,kw OR 'drug therapy'/exp) NOT ('cardiovascular surgery':ab,ti,kw OR 'cardiovascular surgery'/exp OR 'nonsurgical invasive therapy'/exp) NOT ('case report'/exp OR review:ab,ti,kw OR 'systematic review'/exp OR ‘meta analysis’:ab,ti,kw OR 'meta analysis'/exp) NOT (cancer:ab,ti,kw OR neoplasm*:ab,ti,kw OR 'neoplasm'/exp) AND 'article'/it AND ([dutch]/lim OR [english]/lim OR [german]/lim)

Supplemental Table I: Overview of the included studies describing in- and exclusion criteria, population characteristics, determinants and outcomes.

Author, Country, Year	N	Women (%)	Age (years)	Inclusion criteria	Exclusion criteria	Mean LVEF (%)	Determinant	Other ECG parameters measured	Outcome	Prevalence outcome n(%)	Definition of outcome (reference)	Association measure(s)	Outcome stratified by sex?	Sex included in multivariate model?
Boles, Ireland, 2010 (29)	90	53%	46	Newly diagnosed untreated hypertension	Symptoms or signs of HF, ischemic heart disease, arrythmia, diabetes, kidney dysfunction, secondary hypertension, valvular heart disease or hemodynamically active drugs	NR	-	P wave duration, P wave dispersion, ventricular activation time	LVDD	50/90 (56%)	Canadian consensus guidelines (55)	P wave dispersion: 42ms (±1.9) vs 35ms (±2.5) in individuals with vs without LVDD (p-value 0.006). VAT: 39.1ms (±0.3) vs 46.0ms (±0.4)) in individuals with vs without LVDD (p-value <0.0001). E/A and E/e' values only independent determinant of VAT (R^2 = 0.40; regression analysis)	no	no
Dogan, Turkey, 2003 (30)	53	60%	53	Age ≤60 and hypertension	Persistent or permanent AF, BBB, pre-excitation syndrome, LV systolic dysfunction, anti-arrhythmic drug therapy, known structural heart disease (valvular, congenital heart disease, CAD, cardiomyopathy, pericarditis).	NR	-	Maximal P wave duration, minimal P wave duration, P wave dispersion	LVDD	27/53 (51%)	E/A<1, DT>200ms and IVRT>110ms (56)	P wave dispersion 51.5ms (±9.4) vs 41.2ms (±10.6) in individuals with vs without LVDD (p-value <0.01)	no	NA
Eicher, France, 2012 (31)	56	55%	81	Cases: Hospitalization for CHF and/or fulfilling ESC criteria for HFpEF. Controls: Referral for echocardiography due to increased cardiovascular risk or follow-up of stable heart disease	Cases: Persistent AF or atrial flutter. Controls: Severe valvular disease or LVEF<50%	68	-	P wave duration, P-end to R interval.	HFpEF	29/56 (52%)	ESC criteria (15)	No significant differences for P wave duration and P-end to R interval between HFpEF patients and controls	no	NA

Gunduz, Turkey, 2005 (32)	133	50%	58	ECG measurements possible in at least 8 derivations	Previous acute myocardial infarction, thyroid dysfunction, uncontrolled diabetes, chronic liver or renal disease, valvular heart disease, cardiomyopathy, electrolyte disorder, drugs that affect atrial conduction, or alcohol use	57	-	Maximum P-wave duration, Minimum P wave duration, P wave dispersion	LVDD	73/133 (55%)	Grade I-IIII diastolic dysfunction: impaired relaxation, pseudonormalization and restrictive pattern	P wave dispersion 53.9ms (±9) vs 43ms (±9) in patients with LVDD and controls (p-value <0.01)	no	NA
Hayiroĝlu (19) Turkey, 2021		204	24%	ECG and TTE performed at the same day during outpatient clinic visit	LVEF <50%, congenital heart disease, CAD, AF, infiltrative cardiomyopathy valvular disease, pacing, poor quality ECG, any BBB, frequent ventricular arrhythmia, history of PE, history of primary pulmonary hypertension	59	Electrocardiographic index	P wave amplitude in lead V1, R wave amplitude in aVL, S wave amplitude in V1 and R wave amplitude in lead V5	LVDD	86/204 (42%)	normal, indeterminate LVDD, grade I, grade II LVDD (3)	Index: [aVL R wave amplitude * (V1 S amplitude + V5 R amplitude)/P wave amplitude in V1)] ≥ 8.53mV: sensitivity and specificity both 70%, AUC= 0.78. P wave amplitude in V1 ≥ 0,102mV: sensitivity 67%, specificity 60%, AUC= 0.69. R wave amplitude in aVL ≥0.517 mV: sensitivity 62%, specificity 61%, AUC= 0.68. Sum of S wave amplitude in V1 and R wave amplitude in V5: sensitivity and specificity 65%, AUC= 0.68 at cut-off ≥1.85mV.	no	no
Hsu, Taiwan, 2012 (34)	270	43%	57	Referral for echocardiography	Significant aortic or mitral valve disease, AF, inadequate imaging quality.	56	-	LVH	LVDD	~59%	Grade I-IIII diastolic dysfunction: impaired relaxation, pseudonormalization and restrictive pattern (57)	LVH (using Sokolow-Lyon criteria) OR = 3.53 (95% CI: 1.30-9.55) in multivariate analysis.	no	no
Kadi, Turkey, 2015 (35)	72	69%	55	Hypertension, normal coronary angiography	CAD, coronary anomalies, diabetes, systemic disease, renal failure, cardiomyopathy, moderate or severe valve disease, AF, typical LBBB or (incomplete) RBBB, poor imaging quality	NR	-	Fragmented QRS complexes	LVDD	22/72 (31%)	Grade I-IIII diastolic dysfunction: impaired relaxation, pseudonormalization and restrictive pattern (2)	fQRS: OR 3.45 (95% CI: 1.3-9.2) in univariate analysis and OR 7.0 (95% CI: 1.4-35.4) in multivariate analysis.	no	no

Khan, Pakistan, 2016 (26)	300	27%	61	Referral for echocardiography for clinical suspicion of HF	NR	NR	QTc interval	QTc interval	LVDD	180/300 (60%)	Grade I-IIII diastolic dysfunction: impaired relaxation, pseudonormalization and restrictive pattern (58)	QTc interval ≥435 ms: sensitivity, specificity, NPV, PPV and AUC value 71%, 81%, 85%, 65% and 0.82 for LVDD.	no	NA
Krepp, USA, 2014 (25)	185	56%	55	ECG, echocardiography with diastolic function evaluation and CCTA available.	LVEF<45%, significant valvular disease, hypertrophic cardiomyopathy, pericardial constriction, congenital heart disease, pulmonary embolism, primary pulmonary hypertension, atrial or ventricular arrhythmias, LBBB or RBBB, ventricular paced rhythm	64	LVH (upper quartile Cornell product)	PR interval, LVH (Cornell product, Cornell voltage and Solokow Lyon criteria), ST depression, LBBB, RBBB, incomplete block, QT interval	LVDD	105/185 (57%)	Grade I-IIII diastolic dysfunction: impaired relaxation, pseudonormalization and restrictive pattern (16)	PR interval: OR per 10 ms increase 1.15 (95% CI: 1.02-1.13) in univariate model. R wave axis' leftward shift per 10°: OR 1.19 (95% CI: 1.08-1.32) in univariate model. Incomplete BBB present in 10.5% of individuals with LVDD vs 1.3% of individuals without (p-value 0.026). Incomplete BBB: OR 9.3 (95% CI: 1.2-73.1) in univariate model. QRS duration per 10 ms increase not significantly associated with LVDD. No significant differences in QT or QTc. Upper quartile Cornell product: OR 5.91 (95% CI: 2.27-15.42) in multivariate model. Sensitivity, specificity, PPV and NPV of upper quartile Cornell product were 36%, 90%, 83% and 52%.	no	no
Miwa, Japan, 2008 (40)	11	45%	56	Uncomplicated hypertension and initial U wave inversion on ECG	LV wall thickness >12mm	NR	-	initial U wave inversion	LVDD	NA	E/A ratio and deceleration time	Diastolic function parameters normalized and initial U wave inversion disappeared, when blood pressure was lowered by sublingual nitroglycerine administration (E/A and DT significantly improved from 0.7 to 0.9 and 256ms to 192ms).	no	NA
Namdar, Switzerland, 2013 (24)<br><br>Derivation group	164	48%	57	Age>18 years, diastolic function classified according to Nagueh et al. 2009	AF, >1 grade AV block, atrial and/or ventricular pacing, acute ischemia, cardiopulmonary decompensation, LVEF<55%, WMA, poor imaging quality, pericardial effusion, severe valvulopathies, suspected or known familiar forms of cardiomyopathies.	63	PQ interval, T end-P interval, T end-Q interval, T end-P/(PQxAge), T end-Q/(PQx Age)	P wave duration, PQ interval, Pend-Q interval, QT/QTc interval, T peak-T end interval, T end-P interval, T end-Q interval, U wave	LVDD	81/164 (49%)	Grade I-IIII diastolic dysfunction: impaired relaxation, pseudonormalization and restrictive pattern (16)	P wave dispersion, PQ interval and P wave duration longer in LVDD patients. No significant association of QRS with LVDD. QTc interval higher in LVDD (p-value < 0.05). T peak- T end interval: no significant difference. U wave: no significant difference in prevalence.The index T end-P/(PQxAge) showed AUC value, sensitivity, specificity, PPV, NPV and accuracy of 0.96, 90%, 92%, 91%, 90% and 91%. Second index T end-Q /(PQ*Age) showed AUC value, sensitivity, specificity, PPV, NPV and accuracy 0.95, 89%, 94%, 94% 90% and 91%.	no	NA
Validation group	100	60%	45			65	-			50/100 (50%)		Validation of ECG index T end- P/(PQxAge) with AUC value, sensitivity, specificity, PPV, NPV and accuracy of 0.91, 82%, 93%, 93%, 82% and 88%.	no	NA

Nikolaidou, UK, 2017 (33)	2244	51%	72	Suspicion of HF, sinus rhythm	(Ventricular pacing) cardiac device, pregnancy, AF.	NR	-	PR interval, corrected PR interval, QRS interval, QT interval, QTc	HFpEF	1094/2244 (49%)	Signs and symptoms of HF, LVEF≥45% and NT proBNP≥220ng/mL	Median corrected PR interval 168ms (IQR:151-192) vs 163ms (IQR 147-179), p-value <0.001 in HFpEF patients compared to individuals without HF. QTc interval 429ms (IQR: 410-452) vs 418ms (IQR: 401-441) for HFpEF vs no HF. QT interval 406ms (IQR: 384-431) vs 396ms (IQR: 372-416) for HFpEF vs no HFpEF (p-value for both <0.001).	no	NA
Ofman, USA, 2012 (37)	231	61%	65	ECG determined LVH	Age<18 or >90, QRS duration >130 ms, RBBB or LBBB, chronic AF or atrial flutter, ≥2 degree AV block, pacing device, ≥1mm ST elevation or depression, mitral valve disease, iso-electric T waves in lead I, aVL, V5-V6.	62	-	T wave inversion	LVDD	127/189	Grade I-IIII diastolic dysfunction: impaired relaxation, pseudonormalization and restrictive pattern (16)	T wave inversion: OR of 5.6 (95% CI: 2.64-11.29) in multivariate model.	no	yes
Onoue, Japan, 2016 (36)	239	45%	70	LVDD, diagnostic angiography scheduled	NR	62	-	Fragmented QRS complexes	HFpEF	71/239 (30%)	ESC criteria (15) with LVMI from Japanese guidelines (59)	Individuals with fQRS had more often HFpEF (44 vs 22%). fQRS: OR= 3.07 (95% CI: 1.72-5.47) in univariate analysis. OR = 6.75 (95% CI: 1.8-25.3) in multivariate model.	no	no
Palmirie, Denmark, Finland, Iceland, Norway, Sweden, UK, USA, 2006 (38)	791	NR	NR	Blood pressure >160-200/95-115, ECG determined LVH (Cornell voltage product or Sokolow Lyon criteria)	LVEF<40%, stroke or myocardial <6 months prior to inclusion, severe aortic stenosis	NR	-	Strain	LVDD	660/795 (83%)	Grade I-IIII diastolic dysfunction: impaired relaxation, pseudonormalization and restrictive pattern (60)	Strain present in 110 of 791 participants (14%). No significant differences between groups with and without strain with regard to presence and severity of LVDD.	no	yes
Sauer, USA, 2012 (39)	84	47%	52	Referral for exercise doppler echocardiography	Poor imaging quality or doppler tracings, ventricular paced rhythm, atrial arrhythmias	60	-	T peak- Tend, PR interval, QRS duration, QT interval, QTc interval, QRS axis	LVDD	31/84 (37%)	Grade I-IIII diastolic dysfunction: impaired relaxation, pseudonormalization and restrictive pattern (2)	T peak- T end per 10 ms increase: OR 3.9 (95% CI: 1.4-10.7) in multivariate analysis	no	no
Sumita, Japan, 2019 (20)	117	51%	66	Routine echocardiogram and ECG on same day	AF or atrial flutter, history of catheter ablation for atrial arrhythmia, sinus tachycardia, LBBB, pacemaker rhythm, mitral valve stenosis/insufficiency	61	PTFV1, P wave duration, Morris index	PTFV1, P wave duration (≥110 ms, ≥120 ms), Morris index	LVDD	62/117 (53%)	Criteria modified from Nagueh 2016, classifying normal, intermediate, grade I, grade I/II, grade II, or grade III diastolic dysfunction	P-wave duration ≥110ms: sensitivity, specificity, PPV, NPV= 86%, 86%, 93% and 73%. P wave duration ≥120 ms: sensitivity, specificity, PPV, NPV= 34%, 100%, 100% and 41%. PTFV1≥0.04mm*s: sensitivity, specificity, PPV and NPV 27%, 100%, 100% and 38%. Morris index: sensitivity, specificity, PPV and NPV= 13%, 100%,	no	NA

					or history of mitral repair, ASD, pulmonary artery or lung disorder, poor imaging quality or raw data unavailable.							100%, 38%.		
Taha, Egypt, 2014 (23)	140	31%	46	Negative exercise test, false positive exercise test (normal coronary angiography), normal LVEF	LBBB, RBBB, WPW syndrome, drugs influencing QT interval	NR	P wave dispersion, TpTe, QT interval, QTc interval	P wave dispersion, TpTe, QT interval, QTc interval, hump sign during exercise test	LVDD	84/140 (60%)	Diastolic dysfunction with elevated fillings pressures defined as E/e'≥15 (61)	P wave dispersion >0.045ms: sensitivity and specificity 98 and 64%. QTc >0.395ms: sensitivity and specificity 81 and 79%	no	NA
Tan, Singapore, 2019 (28)	1069	45%	55	Controls: healthy participants from Singapore Longitudinal Aging Study and participants with hypertension but without HF. Cases: participating in Singapore HF and Outcomes and Phenotype Study	LVEF<50%, LBBB	NR	Cornell Product	R wave amplitude in aVL, S wave depth in V3, Cornell product	HFpEF	242/463 (52%)	ESC criteria (15)	Cornell Product >1800mm*ms: sensitivity and specificity 40% and 85%. AUC = 0.62	yes	yes
Tsai, Taiwan, 2013 (22)	270	43%	57	Echocardiography after abnormal physical examination, hypertension, or suspicion of CAD or HF	Patients with significant aortic or mitral valve disease, AF or inadequate imaging quality	NR	Corrected P wave dispersion, corrected mean P wave duration, corrected P wave area	Corrected P wave dispersion, corrected mean P wave duration, corrected P wave area	LVDD	89/270 (33%)	Grade I-IIII diastolic dysfunction: impaired relaxation, pseudonormalization and restrictive pattern (57)	AUC values for corrected corrected P wave dispersion, corrected mean P wave duration, corrected P wave area were 0.617, 0.616 and 0.604	no	yes

Wilcox, USA, 2011 (27)<br><br>Derivation group	75	41%	59	Echocardiography because of clinical suspicion of HF	AF or irregular heart rhythm	54	QTc interval, JTc interval	PR interval, QRS interval, QT interval, QTc interval, JTc interval, R-wave axis, left atrial abnormality, LVH, LBBB, RBBB	LVDD	48/75 (64%)	Grade I-IIII diastolic dysfunction: impaired relaxation, pseudonormalization and restrictive pattern (16)	QTc interval ≥435ms: sensitivity and specificity 73% and 74%	no	yes
Validation group	100	46%	52	Referral for outpatient stress echocardiography	NR	59	QTc interval	PR interval, QRS interval, QT interval, QTc interval, R-wave axis, left atrial abnormality LVH, LBBB, RBBB	LVDD	NR	Grade I-IIII diastolic dysfunction: impaired relaxation, pseudonormalization and restrictive pattern (16)	Significant association for LVDD (defined as septal e' <8cm/s) with QTc interval >435 ms	no	NA
Yang, Australia, 2017 (21)	417	53%	71	Age ≥65 years, ≥1 risk factors for HF (e.g. hypertension, diabetes, obesity, previous potentially cardiotoxic chemotherapy, previous history of heart disease, family history of HF)	Known or prior HF or HF symptoms, CAD, moderate valvular heart disease, LVEF<40%, AF, inadequate imaging quality	64	PTFV1, Cornell product, minSTm V5V6	PTFV1, QRS duration, QRS axis, minSTmV5V6, LVH (Cornell voltage, Cornell product and Sokolow Lyon criteria)	LVDD	289/447 (65%)	Grade I-IIII diastolic dysfunction: impaired relaxation, pseudonormalization and restrictive pattern (3,16)	No significant association for QRS duration and LVDD>75th percentile of Cornell product: PPV and sensitivity 77% and 29%. Abnormal PTFV1: PPV and sensitivity= 67% and 36%. Abnormal minSTmV5V6: PPV and sensitivity 67% and 28%.	yes	yes

Abbreviations: ASD, atrial septal defect; AF, atrial fibrillation; CAD, coronary artery disease DT, deceleration time; CCTA, coronary computed tomography angiography; ECG, electrocardiogram; HF, heart failure; IVRT, isovolumetric relaxation time;LVEF, left ventricular ejection fraction; LVH, left ventricular hypertrophy; NA, not applicable; NR, not reported; PTFV1, P wave terminal force in V1; (R/L)BBB, (right/left) bundle branch block; VAT, Ventriclar activation time; WMA, wall motion abnormalities; WPW, Wolf-Parkinson-White

Supplemental Table II: ECG features studied with- and without diagnostic measures

LVDD/HFpEF	Phase	ECG feature	Definition	Study	Cut-off value	Diagnostic value
	Atrial activation	P wave amplitude in V1	Peak of P wave to the iso-electric line of TP interval in lead V1	Hayiroĝlu	≥ 0.102mV	AUC= 0.69, sensitivity= 67%, specificity= 60%
LVDD			P-wave terminal force in lead V1 is the multiplication of the amplitude by duration of the terminal part of the P-wave in lead V1.	Sumita (20)	PTFV1 ≥0.04mm*s	sens= 27%, spec= 100%, PPV=100%, NPV= 38%
				Yang (21)	PTFV1 ≤-4000µV*ms	sens= 36%, PPV= 67%
		Morris Index	Present when P wave negative phase' width and amplitude are both > 1 mm.	Sumita (20)		sens= 13%, spec= 100%, PPV=100%, NPV= 34%
		P wave area	P wave area is the multiplication of the P wave amplitude (mV) by 0.5 P wave duration (ms) in lead II.	Tsai (22)	corrected P wave area > 60 ms*mV	AUC= 0.60, sens= 58%, spec= 56%
		P wave duration	Duration of P wave.	Tsai (22)	corrected P wave duration > 85 ms	AUC= 0.62, sens= 65%, spec= 46%
				Sumita (20)	P wave duration > 110 ms	sens= 86%, spec= 86%
				Sumita (20)	P wave duration > 120 ms	sens= 34%, spec= 100%
				Boles (29), Dogan (30), Gunduz (32)	Boles, Dogan, Gunduz	NR
		P wave dispersion	Difference between longest and shortest P wave recorded from multiple ECG leads.	Taha (23)	P wave dispersion > 45 ms	sens= 98%, spec= 64%
				Tsai (22)	P wave dispersion > 65 ms	AUC= 0.62, sens= 62%, spec= 57%
				Boles (29), Dogan (30), Gunduz (32), Namdar (24)		NR
		PQ- and PR interval	Beginning of P wave until onset of Q or R wave.	Namdar (24)	PQ ≥ 150 ms	AUC= 0.65, sens= 78%, spec= 46%, PPV= 58%, NPV= 68%
				Krepp (25)		NR
	Ventricular depolarization	Ventricular activation time	Time between the onset of Q wave to peak of R wave.	Boles (29)		NR
		LVH	Most common criteria for left ventricular hypertrophy include: 1) Cornell voltage criteria: S in V3 + R in aVL > 28 mm (men), S in V3 + R in aVL > 20 mm (women). 2) Cornell product: (amplitude S in V3+R in aVL)*QRS duration. 3) Sokolow Lyon criteria: S wave in V1 and tallest R wave in V5 or V6 are ≥35 mm, or R wave in aVL ≥11 mm.	Krepp (25)	Cornell product ≥ 1595 mm*ms	sens= 36%, spec= 90%, PPV= 83%, NPV= 52%
				Hsu (34), Yang (21)		NR
		Sum of S wave amplitude in V1 and R wave amplitude in V5		Hayiroĝlu (19)	≥ 1.85mV	AUC= 0.68, sensitivity and specificity= 65%,
		R wave amplitude in aVL	R wave amplitude in aVL	Hayiroĝlu (19)	≥0.517 mV	AUC= 0.68, sensitivity= 62%, specificity= 61%,
		R-wave axis	QRS axis between -30° and +90° considered normal.	Krepp (25)		NR
		QRS fragmentation	Notching in R or S wave of the QRS complex (in absence of (in)complete BBB).	Kadi (35)		NR

		QRS duration	Beginning of Q wave until the end of S wave.	Krepp (25), Namdar (24)		NR
		Incomplete BBB	Left or right BBB pattern with QRS duration 90-120 ms.	Krepp (25)		NR
	Ventricular repolarization	QT interval	Interval between Q wave onset and end of T wave.	Taha (23)	QT > 330 ms	sens= 69%, spec= 64%
		QTc interval	As QT interval decreases when heart rate increases, QT interval is often corrected for heart rate (QTc) by Bazett's formula.	Taha (23)	QTc ≥ 395 ms	sens= 81%, spec= 79%
				Khan (26)	QTc ≥ 435 ms	AUC= 0.82, sens= 71%, spec= 81%, PPV= 65%, NPV= 85%
				Wilcox (27)	QTc ≥ 435 ms	sens= 73%, spec= 74%
				Krepp (25), Namdar (24)		NR
		Strain	Downsloping convex ST segments with inverted asymmetrical T wave opposite to QRS axis in lead V5 and/or V6.	Palmieri (38)		NR
		ST segment deviation	ST segment deviation from J point of at least 20 mV.	Yang (21)		sens= 28%, PPV= 67%
		T wave inversion	At least 1mm inversion of T wave in at least one of the leads I, aVL or V5-V6.	Ofman (37)		NR
		T peak - T end	Interval between peak and end of T wave.	Taha (23)	T peak - T end > 95ms	sens= 76%, spec= 29%
				Namdar (24), Sauer (39)		NR
	Full diastolic period	initial U wave inversion	Initial negative deflection of >0.05 mV in depth in leads with positive U-waves.	Miwa (40)		NR
		U waves	Presence of U waves.	Namdar (24)		NR
		T end - P interval	End of T wave to P wave onset.	Namdar (24)	T end - P ≤311 ms	AUC= 0.82, sens= 79%, spec= 72%, PPV= 74%, NPV= 78%
		T end - Q interval	End of T wave to Q wave onset.	Namdar (24)	T end - Q ≤455 ms	AUC= 0.77, sens= 73%, spec= 73%, PPV= 73%, NPV=73%
	Indexes	T end-P/(PQ*age)		Namdar (24)	(T end-P/(PQ*age) ≥0.0333	AUC= 0.96, sens= 90%, spec= 92%, PPV= 91%, NPV= 90%
		T end-Q/(PQ*age)		Namdar (24)	(T end-Q/(PQ*age) ≥0.0489	AUC= 0.95, sens = 89%, spec= 94%, PPV= 94%, NPV= 90%
		Electrocardiographic diastolic index	R in aVL * (S in V1 + R in V5)/P wave amplitude in V1)	Hayiroĝlu (19)	≥ 8.53mV	AUC= 0.78, sensitivity and specificity= 70%
HFpEF	Atrial activation	P wave duration	See LVDD	Eicher (31)		NR
		PQ- and PR interval	See LVDD	Nikolaidou (33)		NR
		P end to R interval	End of P wave until peak of R wave.	Eicher (31)		NR
	Ventricular depolarization	LVH	See LVDD	Tan (28)	Cornell product ≥ 1800 mm*ms	sens= 40%, spec=80%, AUC=0.62

		QRS fragmentation	See LVDD	Onoue (36)	NR
		QRS duration	See LVDD	Nikolaidou (33)	NR
	Ventricular depolarization	QTc interval	See LVDD	Nikolaidou (33)	NR

Abbreviations: AUC, area under the receiver operating characteristics curve; BBB, bundle branch block; HFpEF, heart failure with preserved ejection fraction; LVDD, left ventricular diastolic dysfunction; NPV, negative predictive value; PPV, positive predictive value; PTFV1, P-wave terminal force in lead V1; LVH, left ventricular hypertrophy, sens, sensitivity; spec, specificity.

Supplemental References:

55. Rakowski H, Appleton C, Chan K-L, Dumesni JG, Honos G, Jue J, et al. Canadian consensus recommendations for the measurement and reporting of diastolic dysfunction by echocardiography. J Am Soc Echocardiogr. 2005;9(5):736–60.

56. Cohen GI, Pietrolungo JF, Thomas JD, Klein AL. A practical guide to assessment of ventricular diastolic function using Doppler echocardiography. J Am Coll Cardiol. 1996;27(7):1753–60.

57. Khouri SJ, Maly GT, Suh DD, Walsh TE. A practical approach to the echocardiographic evaluation of diastolic function. J Am Soc Echocardiogr. 2004;17(3):290–7.

58. Ho CY. Echocardiographic Assessment of Diastolic Function. In: Solomon SD, Bulwer B, editors. Essential Echocardiography: A Practical Handbook With DVD [Internet]. Totowa, NJ: Humana Press; 2007. p. 119–31. Available from: https://doi.org/10.1007/978-1-59259-977-6_6

59. Daimon M, Watanabe H, Abe Y, Hirata K, Hozumi T, Ishii K, et al. Gender differences in age-related changes in left and right ventricular geometries and functions: Echocardiography of a healthy subject group. Circ J. 2011;75(12):2840–6.

60. Wachtell K, Bella JN, Rokkedal J, Palmieri V, Papademetriou V, Dahlöf B, et al. Change in diastolic left ventricular filling after one year of antihypertensive treatment: The losartan intervention for endpoint reduction in hypertension (LIFE) study. Circulation. 2002;105(9):1071–6.

61. Dokainish H, Zoghbi WA, Lakkis NM, Al-Bakshy F, Dhir M, Quinones MA, et al. Optimal noninvasive assessment of left ventricular filling pressures: A comparison of tissue Doppler echocardiography and B-type natriuretic peptide in patients with pulmonary artery catheters. Circulation. 2004;109(20):2432–9.
